# Primary and Secondary Diagnoses of Gambling Disorder and Psychiatric Comorbidity in the Swedish Health Care System—A Nationwide Register Study

**DOI:** 10.3389/fpsyt.2018.00426

**Published:** 2018-09-07

**Authors:** Anders Håkansson, Anna Karlsson, Carolina Widinghoff

**Affiliations:** ^1^Department of Clinical Sciences Lund, Psychiatry, Faculty of Medicine, Lund University, Lund, Sweden; ^2^Malmö Addiction Center, Clinical Research Unit, Malmö, Sweden

**Keywords:** pathological gambling, gambling disorder, psychiatric comorbidity, substance use disorders, patient register

## Abstract

**Background:** Psychiatric comorbidity is common in gambling disorder, a condition with low rates of treatment seeking. There is a paucity of documented nationwide data on gambling disorder and its co-occurring psychiatric comorbidities in the health care system.

**Methods:** This is a nationwide register-based study of all patients aged above 18 years who were diagnosed with gambling disorder (corresponding to pathological gambling, code F63.0, in the ICD-10) in Swedish specialized out-patient health care or in-patient care, from 2005 through 2016. All psychiatric disorders co-occurring with the diagnoses were recorded, along with age, gender and the type of medical specialty.

**Results:** A total of 2,099 patients were included (1,784 in out-patient care and 629 patients in in-patient care), among whom 77 percent were men. Treatment uptake during the study period increased significantly in out-patient care, with an increasing uptake of younger individuals, whereas in-patient treatment uptake remained stable. A co-occurring psychiatric diagnosis was registered in 73 percent of patients, more commonly in females (77 vs. 71 percent, *p* < 0.01). Several diagnostic subgroups were more common in women, with anxiety and affective disorders being the most common subgroups. Prevalence of substance use disorders did not differ with respect to gender.

**Conclusions:** Despite a large gap between probable population prevalence of gambling disorder and the number of treated patients, the number of patients treated in out-patient health care with a gambling disorder diagnosis increased over time, with an increasing treatment uptake in younger individuals. Psychiatric comorbidity is common in gambling disorder patients in the health care system, with a higher prevalence in women.

## Background

Around 0.5 percent of the adult population are known to suffer from a level of problem gambling severe enough to fulfill criteria for a diagnosis (1, 2), hitherto defined as pathological gambling in the International Classification of Diseases (ICD-10) (3) and, since 2013, as gambling disorder in the Diagnostic and Statistical Manual of Mental Disorders (4) and in the ICD-11 (3). Gambling disorder (GD) is known to be associated with severe complications, including suicidal behavior (3) and psychological distress in concerned significant others (5).

Psychiatric comorbidity is common in GD. In a meta-analysis, it was reported that among treatment-seeking patients with GD, a majority of patients suffered from a concurrent psychiatric disorder, including 23 percent suffering from depressive disorder, and as many as 21 percent might also have fulfilled the criteria of an alcohol use disorder, with the corresponding figure for drug use disorders being seven percent (6). Likewise, in patients suffering from a substance use disorder, prevalence rates of problem gambling are known to be considerably higher than in the general population (7). Several studies have also described gender differences in psychiatric comorbidity in GD patients. While GD is considerably more common in men (8–10), women have been reported to be more likely to suffer from concurrent non-substance-related psychiatric disorders, compared to their male counterparts (11–14), whereas concurrent substance use disorders may be more common in men (13, 15, 16).

In contrast to the problem burden associated with GD, it has been described that few people with this disorder actually seek treatment (17–19). Partly, this may be due to patient-specific factors, such as the type and severity of gambling (19), but also feelings of shame, pride or problem denial (20). Treatment-seeking has been described to be associated with a lower degree of problem gambling in patients with GD, and with the opposite in patients with a sub-threshold gambling problem (19). However, barriers to treatment may also be related to actual or perceived problems of availability or dissatisfaction related to how treatment is provided (20). Barriers may also be of organizational or structural nature; routes to treatment may be unclear and the responsibility for the treatment of this disorder may be unclearly defined and under-dimensioned (21, 22). Treatment availability may be limited to a lack of knowledge or confidence in non-specialized settings (23), and gambling as a health concern may be less readily adopted in general practice than many other disorders (24). In addition, there is literature describing a relatively high degree of improvement in motivated individuals without formal treatment, although the actual implications of those findings remain to be studied (25).

While previous aggregated analyses have assessed comorbidity in GD patients in separate clinical settings, there has been a lack of nationwide total population data of GD patients in treatment, with respect to their psychiatric comorbidity (6). For this reason, the present study, using national registry data from Sweden, aimed to describe psychiatric comorbidity, as well as gender and age, in a nationwide material of patients diagnosed with GD, either in specialized health care (typically in psychiatry) or in in-patient hospital treatment. Further, this study also aimed to assess changes in treatment uptake over time, and to what extent GD diagnoses were registered as primary or secondary diagnoses, respectively.

## Methods

This study was carried out in accordance with the recommendations of the Ethics Committee in Sweden, the Swedish ethics legislation, and the national register unit of the Swedish Board of Health and Welfare. The protocol was approved by the Regional Ethics Committee, Lund, Sweden (file number 2016/1104). As the study is a national register study involving non-identified data from the national register unit, no informed consent procedure was required.

The study is a register study using data from the Swedish National Patient Registry (SNPR), which includes in-patient and out-patient treatment episodes in hospital or other specialized medical out-patient treatment (including both psychiatry and other disciplines). The study included all individuals in these registries with an ICD-10 pathological gambling diagnosis (F63.0 in the ICD-10, the International Classification of Diseases and related Health Problems, 10th revision) (3) registered either as a primary diagnosis or as a secondary diagnosis, at any time during the study period. While the terminology has changed in current statistical manuals, the system in use during the study period in the present setting was the ICD-10, which uses the pathological gambling terminology. For the harmonization with current literature, the newer term, GD, was used here. All episodes ranging from 2005 to 2016 were included. The Swedish National Patient Registry (SNPR), which is held by the Swedish Board of Health and Welfare, has been reported to have high validity and coverage (26). During the period studied here, the register has been reported to provide virtually full coverage of in-patient treatment, whereas coverage in out-patient specialist health care has been increasing; for 2005, 2006, and 2007, diagnosis has been missing for 56, 23, and 21 percent of visits in psychiatry, respectively (compared to 16 to 13 percent in somatic health care), although this could potentially mean that for patients with more than one visit during 1 year (27), their likelihood of being captured in the register would show better results. Also, missing data for diagnoses have been decreasing to a 4-percent level in 2016 for specialist out-patient specialist treatment as a whole (27).

Other data available in the present study were gender, age at each treatment episode, date at the start of each treatment episode (and for in-patient treatment the dates of hospitalization and discharge), all other primary and secondary diagnoses appearing concurrently with the gambling diagnosis, and the type of treatment provider (in-patient or out-patient specialized in psychiatry vs. other medical specialties, such as emergency medicine, internal medicine, etc, collapsed into an “other” category for those not belonging to psychiatry). In order to avoid identification of the register data in analyses and reporting, information about the geographical setting was disregarded.

Among all out-patients (*N* = 1,850), 65 were aged below 18 years (12 to 17 years of age), as well as 10 patients in in-patient care. While GD is far from unlikely to occur in adolescents, it could intuitively be suspected in the present setting that some of these younger individuals may be addicted to gaming rather than to gambling. Internet gaming disorder, proposed in the current version of the DSM (DSM-5) (4), cannot be diagnosed according to the ICD-10 used in the present setting, but the terminology used in every-day language in Swedish might mix up gambling and gaming disorder in clinicians with limited experience in behavioral addictions. For this reason, and based on the fact that that gambling is illegal before reaching 18 years of age, it cannot be established with certainty that adolescents included in the present register data fulfill criteria of the actual GD diagnosis. This may be further strengthened by a pronounced gender difference between the out-patient individuals below age 18 (six percent female) and the adults (22 percent female). For these reasons, all individuals aged below 18 at their first treatment episode were excluded from further analyses in the present study. No other exclusion criteria were applied in the study.

The number of new patients was recorded for each year, and concurrent psychiatric disorders were recorded from each of the treatment episodes which included a GD diagnosis. Frequencies of concurrent diagnoses (diagnoses co-occurring with a GD episode) was calculated as the prevalence of a diagnostic group during the study period. Also, it was registered whether the GD diagnosis was ever a primary diagnosis during the study period, and whether it was the primary diagnosis at the first treatment occasion in each type of treatment modality (in-patient and out-patient). Males and females were compared with respect to the presence of each diagnostic subgroup. Diagnoses addressed were, in addition to all diagnoses in the psychiatric (“F”) chapter in ICD-10, also diagnostic codes referring to any self-inflicted intentional or accidental injury or poisoning; attempted suicide (X60-84), accidental intoxications (X40-49), and injuries and intoxications with an unclear intent (Y10-34).

### Statistical methods

For the whole study period, year by year, age distribution, percentage of women and percentage with GD as their primary diagnosis were compared, using an ANOVA (for age) and the chi-square test (for gender and primary diagnosis), respectively. In addition, for every year, significant changes compared to the previous year were calculated, using the Mann-Whitney U test (for age) and the chi-square test (for gender and primary diagnosis), respectively. Prevalence of diagnoses for men and women were compared using the chi-square test.

## Results

A total of 2,099 individuals with an ICD-10 diagnosis of pathological gambling (F63.0) were identified in the national in-patient or out-patient register during the study period. Among them, 77 percent were male (*n* = 1,625). Fifteen percent of individuals (*n* = 314) were diagnosed with GD both in in-patient and out-patient treatment. The proportion of patients with GD as their primary diagnosis increased significantly over time in out-patient treatment (*p* < 0.001), but decreased significantly in in-patient treatment (*p* = 0.001, Tables 1, 2), and a total of 55 percent had GD as their primary diagnosis in at least one of the treatment episodes.

**Table 1:** Treatment uptake for gambling disorder (GD) in specialized out-patient health care in Sweden, 2005–2016.

**Year**	**Number of patients, % of total**	**Mean age (std dev)^1^**	**Female gender, (%)^2^**	**GD primary diagnosis at first occasion (%)^3^**
2005	87	39.5 (11.7)	29	41
2006	92	39.1 (13.3)	22	50
2007	88	35.6 (10.7)	15	41
2008	93	34.8 (12.1)	24	33
2009	121	35.1 (11.2)	19	40
2010	111	35.9 (12.2)	23	46
2011	114	36.5 (12.1)	22	44
2012	116	36.6 (12.6)	17	36
2013	182	35.9 (10.8)	16	53**
2014	194	36.1 (10.9)	19	55
2015	262	35.5 (11.9)	26	56
2016	324	35.0 (10.3)	25	67**
**Total**	**1,784**	**36.0 (11.5)**	**22**	**51**

1 *ANOVA, linearity, p < 0.01, F = 7.84*.

2 *chi-square, linear-by-linear, p = 0.14, chi-2 = 16.07*.

3 *chi-square, linear-by-linear, p < 0.001, chi-2 = 45.61*.

** *p < 0.01 compared to previous year*.

**Table 2:** Treatment uptake for gambling disorder (GD) in in-patient health care in Sweden, 2005–2016.

**Year**	**Number of patients**	**Mean age (std dev)^1^**	**Female gender (%)^2^**	**GD primary diagnosis at first occasion (%)^3^**
2005	54	37.3 (12.4)	33	43
2006	40	36.9 (12.7)	23	38
2007	44	39.1 (14.2)	27	32
2008	43	40.4 (12.3)	21	26
2009	47	37.2 (11.8)	15	19
2010	51	36.9 (11.1)	22	29
2011	58	40.7 (13.4)	22	28
2012	44	41.5 (11.8)	16	23
2013	53	39.7 (14.3)	32	21
2014	71	41.0 (12.6)	18	28
2015	64	39.1 (12.4)	38*	13*
2016	60	36.1 (10.6)	28	23
**Total**	**629**	**38.6 (12.5)**	**25**	**26**

1 *ANOVA, linearity, p = 0.81, F = 0.06*.

2 *chi-square, p = 0.12, chi-2 = 16.48*.

3 *chi-square, linear-by-linear, p = 0.001, chi-2 = 10.69*.

* *p < 0.05 compared to previous year*.

### Changes in treatment uptake

Altogether, the out-patient data demonstrated an increase in the treatment uptake over time (Tables 1, 2), with the lowest number of patients seen in the first year of the study period, and an almost four-fold increase of the number of new patients until the last year of the study period. Likewise, a gradual and significant decrease in age was seen for new entrants into out-patient treatment over time, ranging from a mean age of 39.5 years in 2005 to 35.0 years in 2016 (*p* < 0.01 for linearity). In contrast, the gender distribution was not significantly altered across the study period (*p* = 0.14). In in-patient care, the number of patients annually discharged with a GD diagnosis did not change systematically over time, and the annual mean age of patients (*p* = 0.77) and gender distribution (*p* = 0.12) did not change significantly over time.

### Co-occurring psychiatric disorders

In the whole dataset, a total of 73 percent (*n* = 1,531) had another psychiatric diagnosis and/or self-inflicted injury/poisoning concurrent with the GD diagnosis (Table 3).

**Table 3:** Prevalence of ICD-10 psychiatric comorbidity diagnoses in patients receiving a gambling disorder diagnosis in specialized out-patient treatment or in-patient treatment.

	**All (*N* = 2,099), % (*n*)**	**Out-patient (*N* = 1,784), %(*n*)**	**In-patient (*N* = 629), %(*n*)**
Any co-occurring psychiatric disorder	73 (1,531)	70 (1,242)	90 (566)
Mean age		36.0 (std dev 11.5)	38.8 (std dev 12.5)
Female gender	23 (474)	22 (389)	25 (157)
**DIAGNOSTIC CATEGORIES**			
F0 (mental disorders due to known physiological conditions)	1 (13)	0 (9)	1 (5)
F1 (substance-related disorders)	25 (521)	23 (403)	30 (188)
- Alcohol	17 (356)	15 (270)	22 (138)
- Opioid	1 (29)	1 (24)	1 (7)
- Sedatives	2 (42)	2 (30)	3 (17)
- Cannabis	2 (41)	2 (37)	1 (7)
- Cocaine	0 (11)	0 (9)	0 (2)
- Amphetamine use	1 (21)	1 (16)	1 (7)
- Hallucinogenics	0 (2)	0 (1)	0 (1)
- Tobacco	0 (9)	0 (5)	1 (4)
- Solvents	0 (0)	0 (0)	0 (0)
- Polysubstance	5 (102)	4 (79)	5 (33)
F2 (psychotic disorders)	4 (78)	4 (66)	5 (33)
F3 (affective disorders)	33 (684)	30 (542)	39 (246)
F4 (anxiety, dissociative, stress-related, somatoform and other non-psychotic disorders)	34 (724)	32 (563)	41 (258)
F5 (behavioral syndromes associated with physiological disturbances and physical factors)	3 (60)	3 (53)	1 (7)
F6, excluding F63.0 (disorders of adult personality and behavior)	10 (217)	10 (184)	11 (67)
F7 (intellectual disabilities)	1 (18)	1 (13)	2 (10)
F8 (pervasive and specific developmental disorders)	3 (53)	3 (45)	3 (16)
F9 (behavioral and emotional disorders with onset usually occurring in childhood/adolescence)	10 (210)	10 (186)	8 (50)
Accidental overdose (X40-49)	0 (2)	0 (1)	0 (1)
Suicide attempt (X60-84)	3 (65)	0 (8)	10 (60)
Self-inflicted injury/posioning, unclear intent (Y10-34)	0 (4)	0 (1)	1 (4)

A total of 1,784 individuals (85 percent of all diagnosed individuals) were diagnosed in out-patient care, in an average of 2.8 out-patient episodes where GD had been either the primary or secondary diagnosis throughout the study period (std dev 3.9, median 1, inter-quartile range 1–3, range 1–53, with 54 percent reporting only one episode, and another 17 and nine percent report two or three episodes, respectively). Ninety-eight percent (*n* = 1,749) received their first GD diagnosis in psychiatry during the study period (two percent in somatic health care). Twenty-two percent of patients seen in out-patient treatment were women (*n* = 389), and the mean age at treatment entry in out-patient treatment was 36.0 years (ranging from 18 to 78 years). Seventy percent (*n* = 1,242) were also diagnosed with another psychiatric disorder and/or self-inflicted injury/poisoning. GD was the primary diagnosis in 51 percent (*n* = 906) of the initial out-patient contacts where a GD diagnosis had been registered, and in total, 66 percent of out-patients ever received a primary GD diagnosis in any of the out-patient treatment episodes (Tables 1, 3).

In total, 629 patients (30 percent) received a GD diagnosis in in-patient treatment, with an average of 1.6 in-patient episodes with a GD diagnosis (std dev 2.1, median 1, inter-quartile range 1–1, range 1–31, 76 percent had only one in-patient episode and another 13 and five percent had two or three episodes, respectively). Ninety-four percent (*n* = 591) of patients were diagnosed in psychiatric in-patient health care (and the remaining six percent in somatic in-patient settings). Ninety percent of patients (*n* = 566) were diagnosed with another psychiatric disorder and/or self-inflicted injury/poisoning. Twenty-nine percent received GD as a primary diagnosis in any of the in-patient episodes, and in 23 percent of cases (*n* = 144), the first diagnostic episode during the study period was registered with GD as the primary diagnosis. Twenty-five percent of patients were women (*n* = 157), and the mean age was 38.6 years, with an age range from 18 to 83 years (Tables 2, 3).

### Gender differences

Overall psychiatric comorbidity was significantly more common in females than in males (79 vs. 71 percent, *p* < 0.01). This gender difference was seen in out-patient treatment (77 vs. 68 percent, *p* < 0.01), whereas in in-patient treatment, no gender difference was seen (89 percent in males and 86 percent in females, *p* = 0.28). Women were significantly more likely than men to have a registered affective disorder, an anxiety disorder, an F5 disorder (“behavioral syndromes associated with physiological disturbances and physical factors”), or an F6 disorder (disorders of adult personality and behavior, excluding GD). Other diagnostic categories did not differ between genders, including substance use disorders, among which cannabis was the only separate substance which differed between genders (significantly more commonly reported for men). All diagnostic categories are displayed for both genders in Table 4.

**Table 4:** Comparison of psychiatric comorbidity in men and women with gambling disorder in in-patient or out-patient specialized health care (*N* = 2,099).

	**Men (*n* = 1,625), %(*n*)**	**Women (*n* = 474), %(*n*)**	***P***
F0 (mental disorders due to known physiological conditions)	1 (11)	0 (2)	0.74
F1 (substance-related disorders)	26 (416)	22 (105)	0.13
Alcohol	17 (283)	15 (73)	0.30
Opioid	1 (24)	1 (5)	0.49
Sedatives	2 (31)	2 (11)	0.57
Cannabis	2 (39)	0 (2)	0.01
Cocaine	1 (10)	0 (1)	0.28
Amphetamine use	1 (16)	1 (5)	0.89
Hallucinogenics	0 (2)	0 (0)	0.45
Tobacco	0 (7)	0 (2)	0.98
Solvents	0 (0)	0 (0)	N/A
Polysubstance	5 (85)	4 (17)	0.14
F2 (psychotic disorders)	4 (66)	3 (12)	0.12
F3 (affective disorders)	30 (484)	42 (200)	<0.001
F4 (anxiety, dissociative, stress-related, somatoform and other non-psychotic disorders)	33 (538)	39 (106)	0.01
F5 (behavioral syndromes associated with physiological disturbances and physical factors)	2 (3)	5 (26)	<0.001
F6, excluding F63.0 (disorders of adult personality and behavior)	9 (140)	16 (77)	<0.001
F7 (intellectual disabilities)	1 (12)	1 (6)	0.27
F8 (pervasive and specific developmental disorders)	3 (43)	2 (10)	0.51
F9 (behavioral and emotional disorders with onset usually occurring in childhood/adolescence)	10 (165)	9 (45)	0.67
X40-49 (accidental overdose)	0 (1)	0 (1)	0.35
X60-84 (suicide attempt)	3 (51)	3 (14)	0.84
Y10-34 (self-inflicted injury/posioning, unclear intent)	0 (3)	0 (1)	0.91

## Discussion

The present study is, to the best of our knowledge, the first nationwide description of patients seen in specialized health care for gambling disorder, describing concurrent psychiatric disorders, the distribution of primary and secondary diagnoses, and the changes in treatment uptake for this condition over time. The present study confirms the high rates of psychiatric comorbidity and a higher prevalence of psychiatric disorders in women with GD than in their male counterparts, but also presents prevalence and gender comparisons for separate diagnostic categories.

In the present study, psychiatric comorbidity reached 90 percent for in-patient treatment episodes, compared to 70 percent in the out-patient setting. Previous data has been limited for the comparison of these two settings (6), but the high prevalence of other psychiatric diagnoses in the in-patient setting should be seen in light of the probable requirements for hospitalization in psychiatric settings, where a sole gambling disorder may be unlikely to lead to hospitalization without a comorbid psychiatric condition or a very severe complication affecting daily life. The total prevalence of 73 percent psychiatric comorbidity in the present study can be compared to an extensive literature review addressing comorbidity in samples of GD patients. In that paper, the total prevalence of comorbid disorders was summarized for axis I disorders (representing the large majority of diagnoses discovered also in the present study), ranging from 54 percent to 100 percent in four studies, with a lower 21 percent prevalence reported in one study (6). Also, in the present geographical setting, a recent study from an out-patient facility for gambling disorder patients reported that 58 percent of GD patients fulfilled criteria of another psychiatric diagnosis (14). Thus, the 73 percent prevalence reported here may be within the expected range, although higher than the 58 percent reported in a clinical dataset from the same setting but from a facility aimed specifically for the treatment of GD (Sweden) (14). The psychiatric comorbidity in the present study may be particularly elevated by the fact that patients are included also in cases where GD was a secondary diagnosis, revealed in a setting where the patient is primarily seen for another disorder; this potentially may explain the somewhat higher prevalence of psychiatric comorbidity in the present study compared to a GD-specific clinical unit. Altogether, the present study confirms previous findings of psychiatric comorbidity appearing concurrently in a large proportion, possibly even beyond the majority, of patients seeking treatment for a gambling disorder.

In the present study, 25 percent of patients were diagnosed with a co-occurring alcohol or drug use disorder. This figure is well within the range described in previous clinical studies summarized by Dowling and co-workers, where total substance use disorder comorbidity ranged between 7.5 and 48 percent across studies (6). By far, the most common substance use disorders reported were alcohol use disorders, diagnosed in 17 percent of patients. Again, this figure is within the range previously reported, yet, previous prevalence figures have differed widely, between five and 38 percent across studies and settings (6). The most common diagnostic categories were affective (33 percent) and anxiety disorders (34 percent). The figures in the present study are within the range previously reported; Dowling and co-workers summarized prevalence figures for mood disorders to be from five to 38 percent in most studies, although figures above 80 percent were reported in two studies. In the same review paper, anxiety disorder ranged between four and 48 percent in most studies, with the exception of a 94 percent prevalence reported in one study (6). Altogether, the main psychiatric comorbidities reported in this nationwide dataset were the most expected ones, and at rates comparable to international literature.

In contrast, the comparison of disorders between men and women in the present study were only partially expected. A majority of patients in the present study were men, consistent with previous literature (9, 15, 28), and female patients in the present study were significantly more likely than their male counterparts to suffer from a co-occurring psychiatric disorder (11, 13, 29). This is consistent with previous literature reporting higher rates of comorbidity in females, particularly a higher prevalence of non-substance-related comorbidities in women (14), and in the general population, it has been shown that the association between mental health problems and problem gambling is stronger for women than for men (12), and that women have a more rapid course in the development of a gambling problem (13, 30). Also, specifically, mood and anxiety disorders have been reported to be more prevalent among female problem gamblers than among their male counterparts (12), consistent with the findings in the present study. More surprisingly, however, in the present study, substance use disorders were not significantly more common in men than in women with GD, and for all separate substances except for cannabis, no gender difference was seen. This is in contrast to previous findings where a comorbid substance use disorder has been more prevalent in males (15, 16, 30–32). It is difficult to draw conclusions about the reasons for the lack of association between male gender and substance use disorders in the present study. One speculation may be that the treatment system for gambling disorder in the present setting can be described as immature; specific out-patients services have not yet been developed to a satisfactory extent, and from the low number of diagnosed GD patients in in-patient care, it can be concluded that in-patient psychiatric services conduct far too little screening and diagnostic procedures with respect to problem gambling. Thus, it cannot be excluded that treatment uptake for GD patients is relatively more driven by the comorbid problem, such as a substance-related problem, rather than by the gambling problem, and that treatment-seeking and patterns of treatment offered make women relatively more prone to receive both a GD diagnosis and a substance-related diagnosis. As the treatment uptake for GD increases in the country, and in future studies, it remains to be observed whether the results seen here remain, or whether combined gambling/substance comorbidities evolve toward a more traditional gender distribution.

The finding of an increasing treatment uptake of GD patients in psychiatric out-patient care is interesting, including the finding that over time, an increasing percentage of patients in the out-patient setting were diagnosed with GD as their primary diagnosis. Hypothetically, these findings might indicate that in out-patient psychiatry, GD may be increasingly paid attention to in patients primarily treated for other psychiatric conditions, and where GD is typically registered as a secondary diagnosis. Likewise, the findings might indicate that in the out-patient setting, actual treatment of GD may be developing to an increasing extent in recent years, such that patients are seen and diagnosed for GD specifically, without concurrent comorbidity or where a concurrent comorbidity is less highlighted in the diagnostic process than the GD disorder itself. The latter, despite the large gap between treatment needs and treatment provided in the health care system, might support the overall picture that the attention to GD in the medical sector is increasing.

The increased attention to GD in the health care setting may have several explanations. The highlighting of GD in the DSM-5, where GD is defined as an addictive disorder among alcohol- and drug-related disorders, rather than as an impulse control-related problem (4), may have increased the debate around GD as a condition to screen for and to diagnose in settings where otherwise only alcohol or drug use disorders have been diagnosed, with or without concurrent other psychiatric diagnoses. In parallel to this academic interest in GD, a changing gambling market in the present setting, with a higher degree of online gambling possibly perceived as particularly hazardous (14), may have contributed to an increasing attention to gambling issues in clinicians. Along with this, the increased attention to GD also has been manifested in the opening of units or subunits of clinical facilities working with a focus on GD (14), although thus far to a limited extent.

While the gender distribution did not change significantly over time, the increased treatment uptake in out-patient treatment (although not in in-patient treatment) was followed by a significantly decreasing age in treatment over time. The present study cannot confirm whether this reflects a negative development with gradually younger age in problem gamblers over time, or whether it reflects an improved treatment uptake in younger patients, i.e. intuitively a favorable trend with earlier interventions in patients with problem gambling. In a smaller recent report from treatment-seeking patients in one out-patient gambling treatment facility in Sweden, the median age of patients seeking treatment in 2016–2017 was 31.5 years (14), i.e., markedly younger than in the nationwide data described here over a longer period of time, possibly supporting the trend toward younger age in GD patients seen in treatment. Based on national prevalence data on gambling habits, including at-risk and problem gambling, like in other settings, problem gambling is the most prevalent in younger adults (33). It remains to be understood whether the treatment uptake in out-patient treatment and the trend toward younger age continue, and future studies are needed in order to evaluate this.

The increased treatment uptake in out-patient treatment was not reflected in in-patient data, where the annual number of new GD patients remained stable. This may be seen as surprising and most likely reflects the large gap between the number of GD patients in need for treatment and the actual provision of treatment. Although hospitalization in in-patient psychiatry may not represent a core component of the treatment of a gambling-related problem, the high level of severe complications in GD, including suicidal behaviors (34, 35) is likely to require in-patient care from time to time. Thus, this further points to the need for enhanced screening for and attention paid to problem gambling and GD in settings working with other mental health issues, including in psychiatric emergency units and in-patient wards. Given the low total numbers of patients diagnosed in the country, this is likely to require educational campaigns in psychiatry staff.

Interestingly, the proportion of patients with GD as their primary diagnosis decreased in in-patient care. While this could be interpreted as a tendency for GD patients to be increasingly hospitalized in the context of other psychiatric diseases, these findings might also indicate that the increased attention paid to GD in the out-patient setting does not translate into a higher number of in-patients, but rather may fulfill treatment needs for a growing number of patients in out-patient care, such that hospitalization is needed primarily for patients with dual disorders. Further research is needed in order to clarify how the in-patient setting may or may not be affected over time by the increasing number of GD patients in out-patient treatment.

Despite an increasing number of patients diagnosed in this setting over time, the present study also clearly demonstrates the large gap between the number of patients receiving a GD diagnosis in the hospital system, and the probable number of patients who meet criteria of the disorder in the population. With a point prevalence of 0.5 percent for GD, corresponding to 30,000 to 40,000 adults in the Swedish population, and with assumingly higher lifetime prevalence due to the mobility of individuals in and out of this group (36), the numbers reported in this study indicate that very few patients with GD are diagnosed in the specialized health care system. If in-patient is considered to represent a minority of treatment needs in GD, a treatment uptake of around 300 patients annually in the out-patient setting (as in the most recent year studied here) still means that only around one percent of patients with a current GD receive treatment in specialized health care, and with an even lower percentage given the assumption of a higher number of GD individuals in the population across a 10-year period. In the legislation in the present setting, GD has not been included in the treatment responsibility of the social authorities (which are separated from the health care system where the diagnostic system is used and which provides data to the national register used here). Only recently (January 1st, 2018), GD was included in the regulations of social authorities. While the primary health care system (i.e., the care provided by general practitioners) also is not included in the national registers used in the present study, gambling treatment has not been a major focus in primary care in the present setting, and gambling has only recently been highlighted as a health issue to screen for (37), making it unlikely that a major proportion of treatment uptake in Sweden would have occurred in primary care. Consequently, based on the present data, it can be assumed that the proportion of GD patients seen in the health care system is only little above one percent, clearly demonstrating a large gap between treatment needs and the actual treatment provided for a disorder established as an addictive and thereby psychiatric disorder. At the same time, some recent literature have indicated a relatively high degree of improvement without formal treatment, in problem gamblers with a relatively high degree of motivation for change (25). Thus, a certain proportion of patients may not actively seek, or may not even formally need, treatment, but the size of this proportion is unknown, and even in patients, who self-recover, the harms related to the gambling problem still may be significant. Based on the large treatment gap described in the present study compared to expected numbers of patients in the community, these data clearly indicate the need for policy makers in the medical sector to improve treatment availability for GD. Likely, an expansion of treatment resources for problem gambling also have to involve a higher degree of non-specialist involvement, although such efforts may be associated with barriers (23, 24). Recent data suggest that primary care units also in Sweden may be a potential arena for gambling screening and treatment, although this is so far not established (37).

Consistent with this, the comorbidity demonstrated in the present study can describe co-occurring psychiatric manifestations in patients seen in the specialized health care system, rather than a description of the comorbidity in problem gamblers in the population. It is plausible to believe that these concurrent disorders contribute to the psychiatric treatment contact. However, and despite the possible difficulty in defining the primary and secondary diagnosis, in half of patients diagnosed in out-patient treatment, GD was indeed judged by the responsible physician to be the primary diagnosis at the first treatment episode. The total proportion of out-patients with a concurrent psychiatric disorder was 70 percent, somewhat higher than in a previous report from an out-patient GD unit in the present geographical setting, where 58 percent were diagnosed with another psychiatric disorder (14). At the same time, importantly, these figures demonstrate the need to keep in mind that GD also can develop without significant psychiatric comorbidity, and that pathways to a gambling problem are diverse (38, 39). This strengthens the picture of gambling as a public health issue of its own, representing the predominating and possibly only psychiatric problem in a significant proportion of patients.

It is interesting that although the number of new patients in the out-patient setting increased, the gender distribution was not altered, and although some changes in low numbers were noted from year to year, no significant trend over time could be seen. In Abbott's and co-workers' recent paper on problem gambling in the general population, incidence of new cases of problem gambling was comparable for men and women (33), whereas a large difference in GD prevalence between the genders is consistent with previous literature as described above, and comparable to the recent reporting from one out-patient GD facility in the present setting, although this reflects only one geographical setting and only the most recent years (13). More research is needed in order to observe whether the percentage of women in the present diagnostic database reflects the true diagnostic prevalence in the population, and whether it may change due to changes in gambling patterns in men and women (39).

The present study has some limitations. While the ambition of the present work was to address concurrent psychiatric disorders in people diagnosed with a GD, the present data do not include potential psychiatric disorders diagnosed outside of the context of a concurrent gambling problem. While this has rather ensured the data to cover specifically comorbid, concurrent psychiatric problems, it cannot be excluded that rates of psychiatric comorbidity would be ever higher, if including other potential treatment contacts during the study period. In parallel with this, the present data exclude primary data, where, however, GD is unlikely to have been diagnosed to a large extent, but also excludes treatment offered for GD in social authorities outside the mental health treatment system. The data presented here should be seen in the context of a legislation where the treatment of GD was not included in the formal responsibilities of Swedish social authorities, nor specifically mentioned as a responsibility for the medical system, other than in the sense that all psychiatric disorders—including addictive disorders—may intuitively be addressed in psychiatric and other medical care. Another limitation was the small number of diagnoses of GD in non-psychiatric specialist care is difficult to interpret, as treatment episodes for physical disease are not likely to systematically imply a gambling-related diagnosis, and it can be assumed that GD diagnoses from this setting may represent either a psychiatric background picture somewhat related to the reason for acute treatment, rather than an actual treatment episode for the GD disorder itself.

One limitation is the fact that mainly the out-patient register did not have full coverage, especially not during the very first years of the study period. Thus, the increase in the number of out-patients diagnosed with GD should be seen in the light of this coverage data, but clearly, the increase in the number of GD patients in this setting appears to be markedly steeper than what could be expected from only an increase in register coverage; e.g., the annual number of new patients increased more than three-fold from 2008 to 2016 and it did not increase from 2005 to 2006 and 2007, although the large improvement in coverage was seen specifically in that period (27). Thus, attrition and coverage are not likely to affect the overall study finding of an increased number of GD out-patients and the changes in age and comorbidity seen over time.

The findings of the present study has implications for the health care system in a setting where the treatment uptake for GD traditionally has been low. The close connection between GD and mental health problems calls for screening for gambling problems in settings where psychiatric disorders are treated, but also screening for psychiatric disease in GD treatment settings. Again, psychiatric comorbidity is shown to be somewhat higher in women than in men with GD, and this calls for a particular focus on psychological distress in women with risky gambling behavior. Based on data describing a more rapid development of problem gambling in women, and described as a shorter time from gambling onset to problem behavior compared to men with the same disorder, the need for particular screening of early gambling problems in women with psychological distress may be even more pronounced than in their male counterparts.

In conclusion, the present study is to the best of our knowledge the first study to report treatment uptake for GD and its psychiatric comorbidity in a nationwide material of data from the health care system. The study demonstrates a large gap between the number of gambling patients seen in the health care system and the probable number of problem gamblers in the population, but also an increasing treatment uptake for GD, and a gradually decreasing mean age in out-patients with GD. The present study confirms the picture of a high prevalence of psychiatric comorbidity in these patients, including high proportions of affective and anxiety disorders, as well as substance use disorders. Further, gender differences were seen with respect to several subtypes of psychiatric disorders. Somewhat unexpectedly, male gender was not significantly associated with comorbid substance use disorders. Also, this study demonstrates that a relatively large proportion of patients, primarily in out-patient treatment, are diagnosed primarily with GD without another co-occurring psychiatric disorder.

## Author contributions

AH wrote the largest parts of the manuscript, and was the main responsible of the project. AK carried out parts of the statistical analyses and contributed to the writing of the manuscript. CW contributed with overall knowledge in the research area, and contributed significantly to the writing of the manuscript. All authors approved the final version of the manuscript.

### Conflict of interest statement

The authors declare that the research was conducted in the absence of any commercial or financial relationships that could be construed as a potential conflict of interest.
